# Tracking adipogenic differentiation of skeletal stem cells by label-free chemically selective imaging†
†Electronic supplementary information (ESI) available: CARS microscopy and Oil Red O staining images of SSCs cultured in basal media, and Oil Red O staining images of SSCs from 3 different donors cultured in adipogenic media, imaged with different objectives. See DOI: 10.1039/c5sc02168e


**DOI:** 10.1039/c5sc02168e

**Published:** 2015-09-09

**Authors:** Justyna P. Smus, Catarina Costa Moura, Emma McMorrow, Rahul S. Tare, Richard O. C. Oreffo, Sumeet Mahajan

**Affiliations:** a Department of Chemistry and Institute for Life Sciences , Highfield Campus , University of Southampton , SO17 1BJ , UK . Email: s.mahajan@soton.ac.uk; b Centre for Human Development , Stem Cells and Regeneration , Institute of Developmental Sciences , University of Southampton , SO16 6YD , UK . Email: richard.oreffo@soton.ac.uk

## Abstract

Coherent anti-Stokes Raman scattering (CARS) is a chemically selective label-free imaging technique which is rapidly emerging as a powerful alternative to conventional microscopy in biomedicine. The strength of this imaging approach is the provision of rapid insight into chemical distribution especially of small biomolecules such as lipids. The label-free, non-destructive and non-invasive nature of CARS lends itself for use with stem cells, as labelling or staining will render them otherwise unsuitable for therapy. Isolation, enrichment and characterisation of skeletal stem cells (SSCs) and their progeny is of tremendous significance in regenerative medicine. However, SSCs differentiation into bone, cartilage or fat cell types, is currently assessed using several invasive and, typically, destructive methodologies. Thus, CARS presents an exciting alternative to interrogate the differentiation of SSCs in their natural state. In the current study, we have examined the adipogenic differentiation of SSCs over time using CARS imaging and verified the observed differentiation using molecular analysis of gene expression as well as compared the results to conventional Oil Red O lipid staining. We find that the CARS analysis provides an enhanced resolution and definition of lipid droplets, detectable as early as 24 hours and 72 hours after adipogenic induction. Quantification of the CARS image data sets also showed a change in lipid droplet size distribution during the course of adipogenesis over 14 days. Furthermore, CARS provided a superior and facile approach to monitor changes in SSCs as a result of chemical modulation of adipogenic differentiation. The current studies pave the way for the use of CARS as a powerful chemical imaging tool in therapeutics, regenerative medicine and skeletal stem cell biology.

## Abstract 2

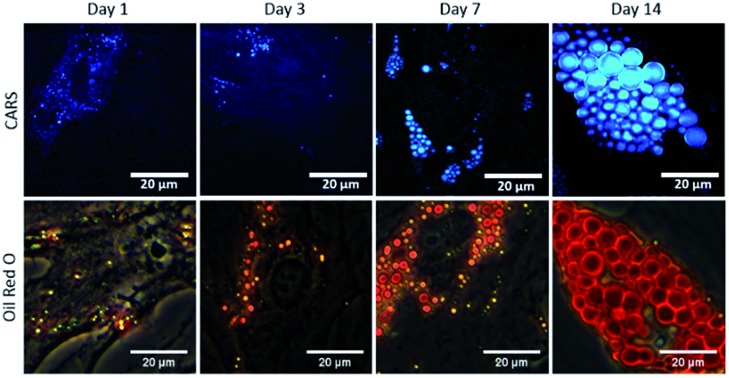
CARS imaging proves to be a powerful, sensitive and label-free tool for studying adipogenesis in skeletal stem cells.

## Introduction

Imaging remains the cornerstone of much of modern biological research providing exquisite resolution of structure and for the study of biochemical processes in cells, tissues and whole organisms. However, the current cellular imaging techniques rely on staining or labelling, with dyes or fluorophores, to visualise target molecules. This is far from ideal as stains are often non-specific and are, typically, only suitable for fixed specimens, while fluorescent labels need to be attached and therefore can interfere with natural processes and, furthermore, can be hampered by photo-bleaching. Consequently the results obtained from such experiments cannot be readily extrapolated to unlabelled cells in a living organism. Vibrational spectroscopy provides an alternative approach, providing chemically selective contrast, generated due to intrinsic vibrations of molecules in their native state, and thus is label-free.^1^

**Fig. 1:**
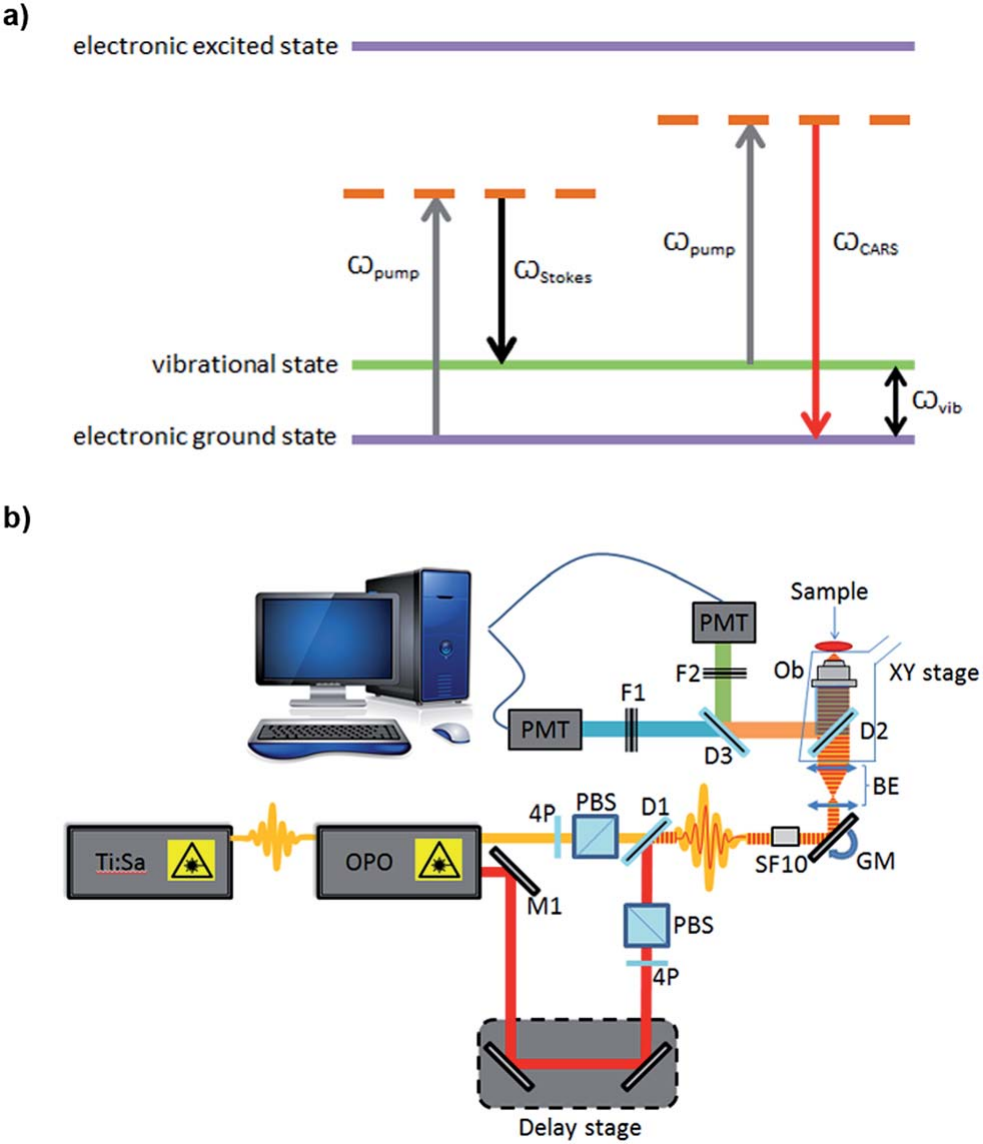
(a) Coherent anti-Stokes Raman scattering phenomenon; (b) schematic of the home-built CARS setup used for imaging. OPO: optical parametric oscillator, PBS: polarizing beam splitter, PMT: photomultiplier SF10, ultrafast laser dispersion compensating prism, BE: beam expander, OB: objective, D: dichroic mirror, F: filter, M: mirror, 4P: half-wave plate, GM: Galvanometer mirrors.

One such vibrational spectroscopic technique is coherent anti-Stokes Raman scattering (CARS), which permits rapid, non-invasive and non-destructive, label-free chemical imaging. In essence, in CARS, the bond vibrations characteristic of a molecule are targeted by using two excitation laser beams (pump and Stokes beam) which are temporally and spatially overlapped.^2^,^3^ In CARS, the pump beam (at frequency *ω*_pump_) and a Stokes beam (at *ω*_Stokes_) combine to yield an anti-Stokes signal at frequency *ω*_CARS_ = 2*ω*_pump_ – *ω*_Stokes_. When the frequency difference between the pump and the Stokes beams matches the frequency of a vibrational mode of the molecule of interest, vibrational coherence is induced. The outcome of this process is a dramatically enhanced anti-Stokes Raman signal that is the foundation for the increased vibrational contrast and sensitivity achieved in CARS which leads to rapid video-rate imaging with <10 μs dwell times.^4^–^6^ CARS signals can be up to 9 orders of magnitude stronger than conventional Raman scattering.^7^,^8^ A schematic showing the energy levels involved in the CARS process is presented in Fig. 1a. Furthermore, as CARS is multiphoton it has natural z-sectioning ability enabling 3D imaging.^9^ Typically, near-infrared wavelengths are used allowing deep penetration into the sample and therefore thick tissues can be imaged with ease.^4^ Consequently CARS offers a chemically selective label-free technique for cells, tissues and organisms in biology and medicine.^10^

In biomedicine, chemically selective, label-free imaging is crucially required in the area of stem cells and regenerative medicine given the requirement for original (un-labelled/un-stained) samples for *in vivo* application in humans. Over the last decade, stem cell-based therapies have come to the fore for tissue repair and skeletal regeneration,^11^ necessitating the development of appropriate technologies to enable stem cell characterisation and thus enhance therapeutic applications. While CARS imaging has been applied to stem cells,^12^,^13^ its application in human skeletal regeneration studies, to date, remains limited. Current available techniques to characterise and monitor the ability of human skeletal stem cells (SSCs) to form cartilage, bone and fat, are invasive and unsuitable for time-course studies. In the only published study using CARS with SSCs, Schie and co-workers performed a primordial assay using adipocytes from human bone marrow stem cells and imaged the lipid droplets after 21 days of differentiation, with the primary objective to improve the simultaneous imaging in forward- and epi-CARS microscopic system.^14^ Neither the differentiation process of SSCs nor the effect of chemical modulation was studied, nor was any CARS based analysis performed.

In the current study, we show the application and advantageous use of label-free CARS imaging to study early adipogenic differentiation of human SSCs. In the absence of exogenous labels, we monitor lipid droplet formation, to assess temporal changes in adipogenesis, subsequently correlated with gene expression analysis. The current studies also demonstrate the potential of CARS to study the effect of growth factors and inhibitors on the SSC differentiation process and the resolution afforded by this technique to detect ultra-small lipid droplets. The current work establishes the utility of chemically-selective CARS imaging for skeletal stem cell biology and potential wider applications in hard and soft tissue characterisation in the field of regenerative medicine.

## Materials and methods

All reagents were supplied by Sigma-Aldrich (UK) unless otherwise stated.

### Ethics statement

The research work was conducted under ethical approval LREC 194/99/1 (National Research Ethics Service – Southampton and South West Hampshire Research Ethics Committee), with informed consent from each donor or their next of kin.

### Bone marrow preparation and STRO-1+ isolation

Bone marrow samples were collected during routine total hip replacement surgery. After cell extraction from bone marrow, red blood cells were removed using Lymphoprep™ (Lonza, Switzerland). The STRO-1+ fraction was isolated from human bone marrow stromal cells by magnetic activated cell sorting using STRO-1 antibody hybridoma (a robust marker for enrichment of SSCs).^15^ SSCs were cultured in basal media (α-MEM supplemented with 10% (v/v) foetal bovine serum and 1% (v/v) penicillin–streptomycin, Lonza), and maintained in a humidified chamber at 37 °C and 5% CO_2_. Cells were harvested at 85% confluence using a 0.25% trypsin/EDTA solution (Lonza).

### *In vitro* differentiation into adipocytes

Adult SSCs were seeded into cell culture plates at a density of 3000 cells cm^–2^ in basal media, and cultured for 6 days. Fresh adipogenic media (basal media supplemented with 100 nM dexamethasone, 0.5 mM 3-isobutyl-1-methylxanthine, 3 μL mL^–1^ 100× ITS liquid media supplement and 1 μM rosiglitazone) was added to induce SSCs adipogenic differentiation. Cells treated with basal media served as control. Adipogenic/basal media was changed every 3–4 days. Cells were characterised by Raman spectroscopy, CARS, gene expression analysis and Oil Red O staining, at days 0, 1, 3, 7 and 14, following basal/adipogenic media addition. For Raman spectroscopy and CARS imaging the cells were fixed using 4% (w/v) paraformaldehyde, which is known not to substantially affect lipid content.^16^

### Raman spectroscopy

For Raman spectroscopy, SSCs were cultured for 14 days, in both basal and adipogenic media, on sterilised glass coverslips. Cells were washed with Dulbecco's phosphate buffered saline (PBS) and fixed in 4% (w/v) paraformaldehyde for 30 minutes, at room temperature. Following fixation, cells were washed with excess PBS, and Raman spectra was obtained using a Renishaw® inVia Raman microscope with a 633 nm laser and a Leica 63× (NA:1.2) water immersion objective, in combination with WiRE 3.4 software. For each spectrum, 3 accumulations were collected using a 1200 lines per mm grating, 0.6 mW laser power at the sample, and an exposure time of 10 s. Cosmic ray artefacts were removed using WiRE 3.4. Before plotting the spectra they were processed using IRootLab, a MATLAB based toolbox for vibrational spectroscopy. Wavelet denoising and baseline correction was carried out by fitting a 9^th^ order polynomial in IRootlab.^17^,^18^

### CARS

For CARS imaging, SSCs were cultured on sterilised glass coverslips. Cells were cultured for 1, 3, 7 and 14 days. While in human skeletal stem research cells are usually assessed after 2–3 weeks,^19^ in this work we wanted to demonstrate the advantageous use of CARS imaging for early detection of adipogenic differentiation. Hence, early time points of 1, 3 and 7 days were selected in addition to day 14. After culturing for different time points, cells were washed with PBS and fixed in 4% (w/v) paraformaldehyde for 30 minutes, at room temperature. Cells were washed with excess PBS, and images were captured using a home-built system (Fig. 1b). A Chameleon Ultra titanium sapphire Ti:Sa laser (Coherent, UK) was used to generate a beam which was then split into two. One beam was used as the pump beam, and the other beam was used to drive an Optical Parametric Oscillator (OPO) (APE Berlin, Germany) to generate the Stokes beam. Both the beams were spectrally compressed (to achieve ∼50 cm^–1^ spectral resolution), spatially and temporally combined and collinearly input into an inverted microscope (Nikon Ti-U), and the resulting epi (back-scattered) CARS signal was detected. Epi-detection was used as this approach minimises the non-resonant background signal that is present during imaging. For imaging lipids in the cells, the C–H stretching mode at 2845 cm^–1^ was targeted, using a pump beam wavelength of 835 nm and the OPO beam of 1097 nm. Lipids were visualised as bright areas on the images. Each sample was imaged using a 40× objective, with a 2× optical zoom using galvanometric scanning, and the acquisition time was 16 ms per line for taking a 512 × 512 pixel image; thus the pixel dwell time was ∼30 μs. The total incident power on the sample was 30 mW with 20 mW in the pump and 10 mW in the Stokes beam. Four images were taken from each coverslip (12 images for each time point). ImageJ was used to process the images. The ratio of the lipid area to cell area was quantified, as well as the size and number of single lipid droplets, during the time-course experiment. The non-resonant background from the cell area was used as the reference for demarcation of lipid areas for quantification.

### RNA extraction and cDNA synthesis

Cells were disrupted and homogenised in a lysis buffer (Buffer RLT Plus, from Qiagen, Netherlands), for each different time-point. Total RNA was isolated using Qiagen RNeasy Plus Mini Kit according to the manufacturer's instructions. The RNA for each sample was quantified using a Nanodrop UV-vis spectrophotometer, and dilutions were made in order to have the same amount of RNA for each sample in the experiment. cDNA synthesis was performed using the SuperScript® VILO™ cDNA Synthesis Kit and Master Mix, following the manufacturer's instructions.

### Quantitative real-time polymerase chain reaction (qPCR)

Relative quantification of gene expression was performed with qPCR using an ABI Prism 7500 detection system (Applied Biosystems). Samples were run in triplicate, containing 1 μL cDNA, 10 μL Power SYBR Green PCR Master Mix (Applied Biosystems), and 500 nM of each primer (primers used for qPCR are shown in Table 1). Thermal cycler conditions were programmed with an initial activation step at 50 °C for 2 minutes and 95 °C for 10 minutes, followed by 40 cycles of 95 °C for 15 seconds and 60 °C for 60 seconds. A melt curve stage was also included with 95 °C for 15 seconds, 60 °C for 1 minute, 95 °C for 30 seconds, and finishing with 60 °C for 15 seconds. The relative quantification of gene expression was determined using the 2^–ΔΔCt^ method. All data were compared to day 0 values, and normalised to *ACTB* expression.

**Table 1:** Primers used for qPCR (F: forward and R: reverse)

Transcript	Abbreviation	Primer sequence (5′–3′)
Beta-actin	*ACTB*	F: GGCATCCTCACCCTGAAGTA
		R: AGGTGTGGTGCCAGATTTTC
Peroxisome proliferator-activated receptor gamma	*PPARG*	F: GGGCGATCTTGACAGGAAAG
		R: GGGGGGTGATGTGTTTGAACTTG
Fatty acid binding protein 4	*FABP4*	F: TAGATGGGGGTGTCCTGGTA
		R: CGCATTCCACCACCAGTT
Alkaline phosphatase	*ALPL*	F: GGAACTCCTGACCCTTGACC
		R: TCCTGTTCAGCTCGTACTGC
Alpha-1 type I collagen	*COL1A1*	F: GAGTGCTGTCCCGTCTGC
		R: TTTCTTGGTCGGTGGGTG

### Oil Red O staining

SSCs cultured for different time points (0, 1, 3, 7 and 14 days), were washed with PBS and fixed in Baker's formal calcium solution, containing 1% (w/v) calcium chloride and 4% (v/v) formaldehyde, for 25 minutes at room temperature. After fixation, cells were rinsed with 60% (v/v) isopropanol, and stained using double-filtered Oil Red O solution for 15 minutes. To prepare the staining solution, a saturated Oil Red O solution in isopropanol was mixed with dH_2_O, to a final ratio of 3 : 2. Cells were washed three times with excess dH_2_O, and images were captured using a Carl Zeiss Axiovert 200 inverted microscope, AxioCam HR (colour) digital camera, and the software package AxioVision 4.7.0.0.

### Effect of dexamethasone and bisphenol A diglycidyl ether in adipogenesis

SSCs were seeded and cultured as detailed for adipogenic differentiation. Dexamethasone concentration in adipogenic media was modified to either 50 nM or 200 nM, to evaluate the effect of dexamethasone on adipogenesis. Adipogenic media was changed every 3–4 days, and cells were characterised at day 14. Similarly, bisphenol A diglycidyl ether (BADGE) was chosen as an inhibitor of the adipogenesis.^20^ To assess its effect in adipogenic differentiation of SSCs, fresh adipogenic media was supplemented with 7.5 μM BADGE. Adipogenic media with BADGE was changed every 3–4 days, and cells were characterised at days 1, 3, and 5.

### Statistical analysis

The results are presented as the mean ± standard deviation. Statistical analysis was performed using IBM® SPSS® Statistics version 21.0 (IBM Corporation, Armonk, NY, USA). The Mann–Whitney test was performed to compare two independent groups. All experiments were performed using at least three separate bone marrow samples, from three different patients. Differences were considered to be statistically significant at *P* < 0.05.

## Results and discussion

In this work, we have studied the differentiation of skeletal stem cells (SSCs) into adipocytes and the effect of different chemical factors using the non-invasive, non-destructive and chemically selective label-free technique of CARS.

**Fig. 2:**
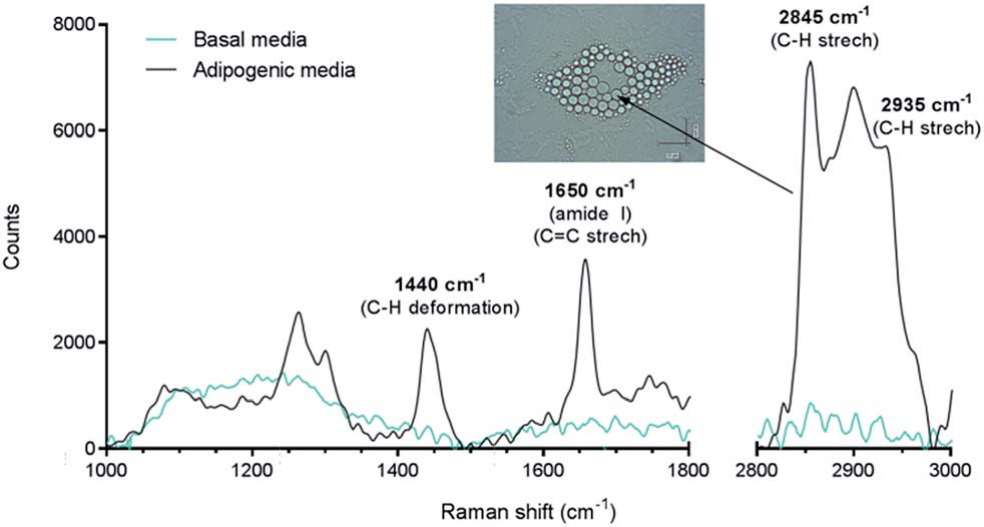
Raman spectra of differentiated (adipogenic media) and undifferentiated (basal media) SSCs (laser 633 nm, 0.6 mW power), and bright field image of lipid droplets that yielded the Raman signal. The spectrum from cells cultured for 14 days in adipogenic media was acquired by directly focussing on the lipid droplets. The prominent C–H stretching mode peak at 2845 cm^–1^ was targeted to image lipids using CARS microscopy.

At the outset Raman spectroscopy was carried out to establish the chemically selective vibrational frequency for imaging lipids in SSCs by CARS. Therefore Raman spectra were obtained by focussing on large lipid droplets in differentiated adipocytes. A representative spectrum is shown in Fig. 2. SSCs were cultured for 14 days in adipogenic media in order to visualise lipid droplets and obtain their Raman spectra, since, unlike CARS, signals in Raman spectroscopy are weak. Nevertheless, the spectra were characteristic and reproducible for different lipid droplets acquired in different cells. A strong peak at 2845 cm^–1^ corresponds to the CH_2_ symmetric stretching frequency, indicating the predominant presence of lipids. Additional peaks at 1440 cm^–1^ and 1650 cm^–1^ correspond to the C–H deformation and C=C stretching vibrations, respectively,^21^ and symmetric CH_3_ stretching modes at 2935 cm^–1^ confirm the assignment to the presence of lipid droplets in adipocytes.^22^ It is also known that 1650 cm^–1^ and 2935 cm^–1^ peaks are associated with amide I and CH_3_ stretching in proteins. As the content of unsaturated fatty acids in lipid droplets is known to be variable,^23^ these peaks likely originate from adipose differentiation-related protein (ADRP) and caveolin 2β, which are proteins dominantly associated with lipid droplets and their membranes.^24^,^25^ These peaks were not observed in undifferentiated SSCs (cultured in basal media, no lipid droplets) under the acquisition conditions. When compared to traditional characterisation techniques, this Raman-based spectral analysis thus allows identifying, locating and comparing molecular composition between different samples. Nevertheless, the Raman spectra of the samples confirmed the strong presence of the C–H stretching vibration characteristic of lipids,^26^ and established the suitability of using the 2845 cm^–1^ vibrational frequency to image lipid droplets in SSCs and was subsequently used for CARS imaging.

**Fig. 3:**
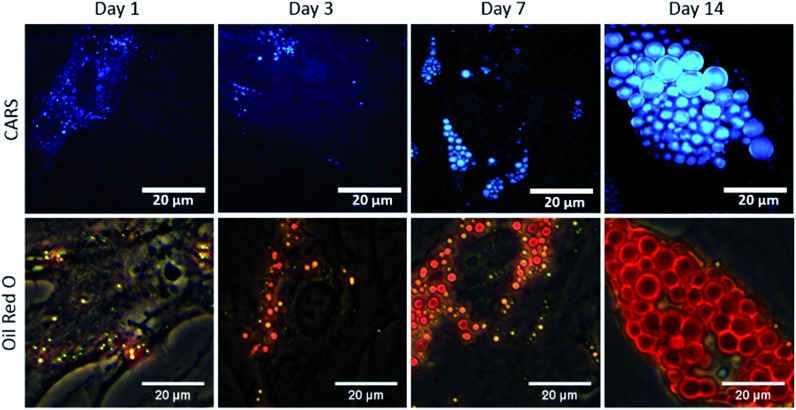
Comparison of label-free CARS imaging and Oil Red O staining to assay adipogenic differentiation of skeletal stem cells (SSCs). SSCs were cultured in adipogenic media for 1, 3, 7 and 14 days. Scale bars correspond to 20 μm.

To study the temporal evolution of their differentiation, SSCs were cultured in adipogenic and basal media, and temporal changes observed using CARS over 14 days. Adipocytes are readily identified by the accumulation of lipid droplets through the differentiation process.^27^ The C–H stretching vibration signal at 2845 cm^–1^ was monitored by CARS microscopy by tuning the pump and OPO beams to 835 nm and 1097 nm, respectively. Representative CARS images acquired at day 1, 3, 7 and 14 are shown in Fig. 3. Additionally, adipocytes were imaged at the same time-points using conventional Oil Red O staining of lipids. Both CARS and Oil Red O staining showed similar lipid droplet distributions over the time-points studied. CARS microscopy and Oil Red O staining images of SSCs cultured in basal media, which show very few lipid droplets and do not show an increase over time, are shown in Fig. S1 (see ESI†). Images taken at different magnifications with Oil Red O staining are shown in Fig. S2.1–3 (see ESI†) demonstrate cell growth over time over different fields of view. Overall these results demonstrate that while there is good correspondence between both methodologies, several advantages of CARS over Oil Red O staining are clearly apparent. CARS provided a chemically-selective and label-free approach, in contrast to the application of Oil Red O staining that required a much more laborious process and is subject to variability depending on preparation conditions.^28^ Most significantly, in terms of performance, CARS displays a higher sensitivity towards detection of small lipid droplets (blue) observable after only 24 hours and 72 hours, but poorly visualised using Oil Red O staining. Moreover, at low levels of lipid droplet presentation, such as at day 1, the generation of artefacts due to Oil Red O crystal deposition and non-specific binding cannot be ruled out. These results demonstrate the feasibility of CARS as a label-free chemical imaging tool to monitor SSCs differentiation in a temporal manner, thereby facilitating detection of early adipogenic differentiation.

**Fig. 4:**
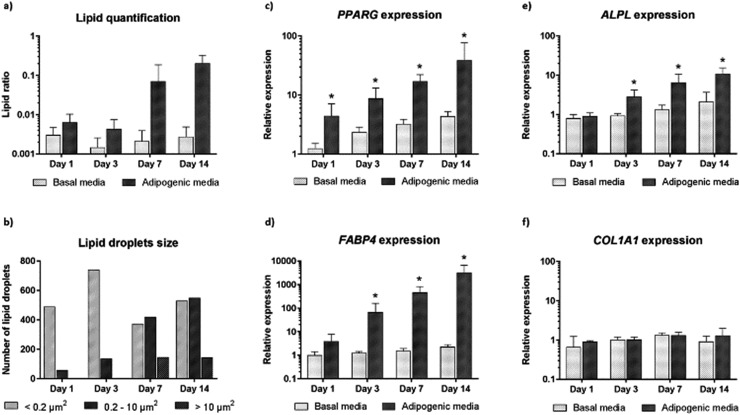
Quantitative analysis of CARS images and comparison with gene expression profiles. (a) Ratio of lipid area to cell area and (b) size of lipid droplets in skeletal stem cells cultured in basal and adipogenic media, for 1, 3, 7 and 14 days. Expression of (c) *PPARγ* (d) *FABP4*, (e) *ALPL* and (f) *CoL1A1* in skeletal stem cells cultured in basal and adipogenic media, for 1, 3, 7, and 14 days. Relative expression was normalized to *ACTB*, and day 0 values were set to an expression of one. In all of the above the average of three independent patient samples is plotted and error bars represent standard deviation. *P < 0.05, calculated using Mann–Whitney test.

We further carried out image analysis to quantify the increase in lipid droplets and change in lipid size. Lipid droplet quantification observed using CARS is shown in Fig. 4a. As differentiation proceeds, adipogenesis leads to increased lipid accumulation in contrast to control cultures (undifferentiated cells maintained in basal media). We further analysed the presence and change in the number of lipid droplets within the cultures (Fig. 4b). The presence of only small lipid droplets (<0.2 μm^2^) was detected at day 1. However, by day 3, small (<0.2 μm^2^) and medium (0.2–10 μm^2^) lipid droplets had increased in number. Finally, at day 7 and day 14, it was possible to detect a decrease in the number of small lipid droplets, and an increase in the number of medium (0.2–10 μm^2^) and large (>10 μm^2^) lipid droplets. The increase in size of small and medium lipid droplets, respectively, points to early changes during SSC differentiation to adipocytes detected by CARS before they are fully formed.

In order to confirm adipogenic differentiation and correlate to the CARS imaging, we also examined the gene expression profile of characteristic adipogenic genes (*PPARγ* and *FABP4*) and non-specific (osteogenic) genes (*COL1A1* and *ALPL*) at corresponding time-points. The expression profile in basal and adipogenic media is shown in Fig. 4c–f. *PPARγ* expression, a nuclear receptor gene known to regulate the development of adipose tissue,^29^ increased significantly during differentiation into adipocytes (Fig. 4c). From day 1 to day 14, large variation in *PPARγ* relative expression was observed in SSCs cultured in adipogenic media, and expression levels were 39-fold higher at day 14. The increase in *PPARγ* expression, a key regulator gene in adipogenesis, appears to correlate with the development and increase of lipid droplets in SSCs cultured in adipogenic media, as shown in Fig. 3. In addition, we assessed *FABP4* expression (encodes fatty acid binding protein in adipocytes during adipogenic differentiation) known to be regulated by *PPARγ* agonists, insulin and fatty acids.^30^ Comparing the values between SSCs cultured in adipogenic media with day 0 cultures, *FABP4* displayed a 66-fold increase in expression at day 3, a 458-fold increase at day 7, and 3180-fold increase at day 14. *FABP4* expression exponentially increased compared to undifferentiated cells (Fig. 4d). *ALPL* and *COL1A1* expression was also assessed as a control (Fig. 4e and f).^31^

Adipogenesis and osteogenesis share the same pathway in the early differentiation cascade,^32^ and *ALPL* is believed to be involved, in an as yet undetermined manner, in the control of adipogenesis – *ALPL* is associated with intracellular lipid droplets.^33^,^34^ In Fig. 4e, a modest increase in *ALPL* relative expression during adipogenesis was observed which suggests that *ALPL* also has a role in the adipogenic differentiation of SSCs. However, *COL1A1* expression was unchanged over time in differentiated and undifferentiated cells as expected. Such molecular analysis to monitor gene expression, although destructive and ensemble based, remains currently a key sensitive tool to monitor phenotypic changes in cells. The CARS imaging results correlated closely with the gene expression studies, affirming the utility of CARS in skeletal stem cell biology.

**Fig. 5:**
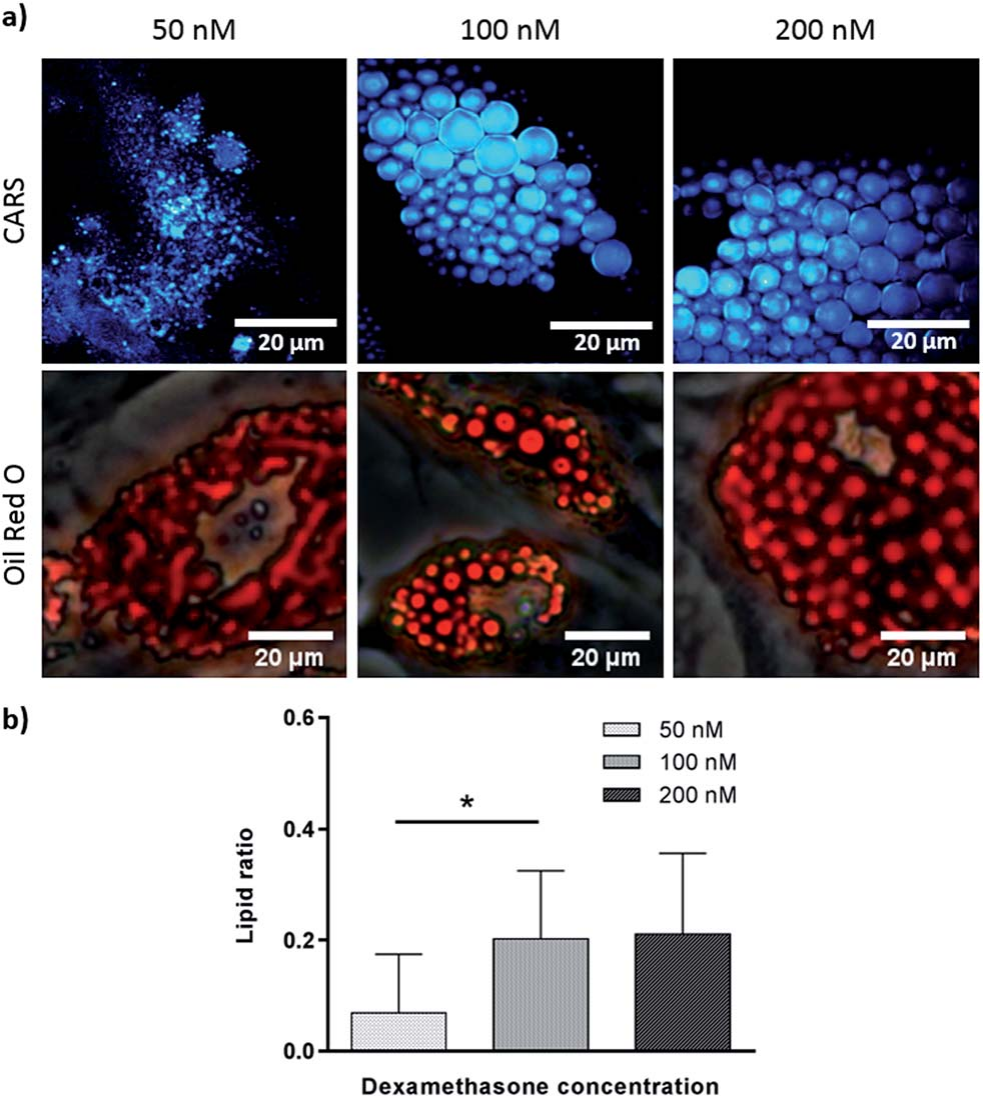
Skeletal stem cells cultured for 14 days in adipogenic media with different concentrations of dexamethasone (50 nM, 100 nM and 200 nM). (a) Comparison of label (Oil Red O staining) and label-free (CARS) imaging techniques to assay adipogenesis. Scale bars correspond to 20 μm. (b) Quantitative analysis of lipid storage. Data represent an average of the ratio of lipid area to cell area of three independent patient samples, and error bars represent standard deviation. *P < 0.05, calculated using ANOVA comparatively to adipogenic media (100 nM dexamethasone).

**Fig. 6:**
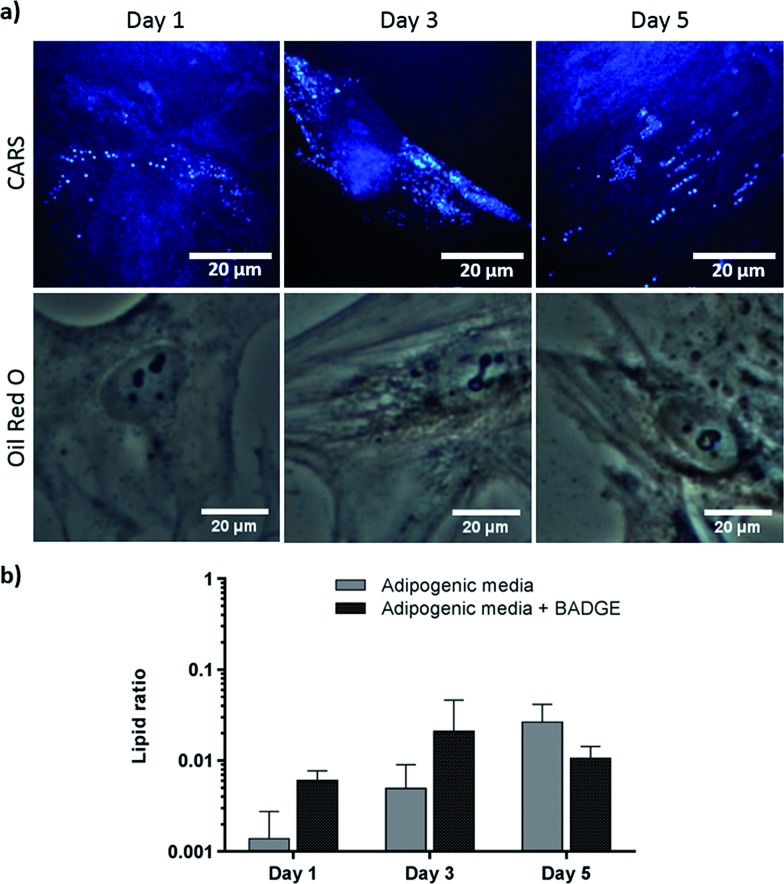
Skeletal stem cells cultured for 1, 3 and 5 days, in adipogenic media supplemented with 7.5 μM BADGE. (a) Comparison of label (Oil Red O staining) and label-free (CARS) imaging techniques to assay adipogenesis. Scale bars correspond to 20 μm. (b) Quantitative analysis of lipid storage. Data represent an average of the ratio of lipid area to cell area of three independent patient samples, and error bars represent standard deviation.

To verify further the application of label-free CARS in monitoring the temporal evolution of SSCs differentiation, we explored its use to investigate the effect of chemical compounds typically used to modulate the differentiation process. Here we studied the effect of dexamethasone (Fig. 5) and bisphenol A diglycidyl ether (BADGE) (Fig. 6) on adipogenic differentiation. SSCs were cultured for 14 days in adipogenic media with different dexamethasone concentrations (50–200 nM). Dexamethasone modulated adipogenesis evidenced by representative CARS and Oil Red O staining images of differentiated SSCs (Fig. 5). Lipid droplet accumulation was significantly larger at 100 nM and 200 nM dexamethasone concentrations compared to 50 nM. Although changes are observed with both CARS and Oil Red O staining, at the lower concentration of 50 nM, lipid droplets are smaller and more effectively (higher definition) imaged by CARS microscopy. We further used BADGE to demonstrate the effect of an inhibitor on adipogenesis.^20^ SSCs were treated with 7.5 μM BADGE, and cells were monitored over 5 days (Fig. 6). The induction of lipid-rich organelles at day 3 can be seen in CARS images. The inhibition of adipogenesis can be observed by CARS with a decrease in lipid expression on day 5. This is summarised in the bar graph in Fig. 6b which compares the results of the inhibitor with those on untreated adipogenic cells. It shows that on treatment with BADGE the lipid droplets increase and then decrease, while they continue to increase in its absence as shown earlier in Fig. 3 and Fig. 4a. Most importantly the conventional Oil Red O staining procedure is unable to pick up any of these changes. This clearly establishes the superiority of CARS in imaging minute changes during differentiation as a result of modulation by chemical treatments.

## Conclusions

To date, SSCs differentiation has been characterised and studied using invasive methods; therefore, a reliable non-invasive, non-destructive and label-free (stain-less) imaging technique is urgently required to evaluate SSC fate and function. We demonstrate that the chemically selective technique of CARS imaging provides an ideal alternative to monitor SSC differentiation. Lipid accumulation within adipocytes can be visualised earlier, at higher definition and resolution, compared to conventional Oil Red O staining approach. We further demonstrate that the modulation of human SSC differentiation using different chemical cues can be effectively assessed by CARS. Thus these studies confirm the robust potential of a CARS-based assay to monitor and understand the effect of physicochemical parameters on stem cell differentiation. The results establish the value of using CARS as a label-free chemical imaging technique for skeletal stem cell biology with implications therein for wider stem cell biology, regenerative medicine and therapeutics.

## Disclosure

The authors have no potential conflicts of interest.

## Supplementary Material

Supplementary information
