# Supplementary material for: Tracking adipogenic differentiation of skeletal stem cells by label-free chemically selective imaging
Source: Chem Sci. 2015 Sep 9;6(12):7089–96. doi: 10.1039/c5sc02168e (PMC5951131; doi:10.1039/c5sc02168e)
Supplement: Supplementary file 1 [file SC-006-C5SC02168E-s001.pdf]

Electronic Supplementary Material (ESI) for Chemical Science.
This journal is © The Royal Society of Chemistry 2015

Electronic Supplementary Information (ESI)

Tracking adipogenic differentiation of skeletal stem cells by label-free chemically selective imaging

Justyna P. Smus,[a#] Catarina Costa Moura,[ab#] Emma McMorrow,[b] Rahul S. Tare,[b] Richard O. C. Oreffo,[b]* Sumeet Mahajan[a]*

[a]*Department of Chemistry and Institute for Life Sciences, Highfield Campus, University of Southampton, SO17 1BJ, UK.*
[b]*Centre for Human Development, Stem Cells and Regeneration, Institute of Developmental Sciences, University of Southampton, SO16 6YD, UK.*

Correspondence email: s.mahajan@soton.ac.uk, richard.oreffo@soton.ac.uk
[#]These authors contributed equally to this work.

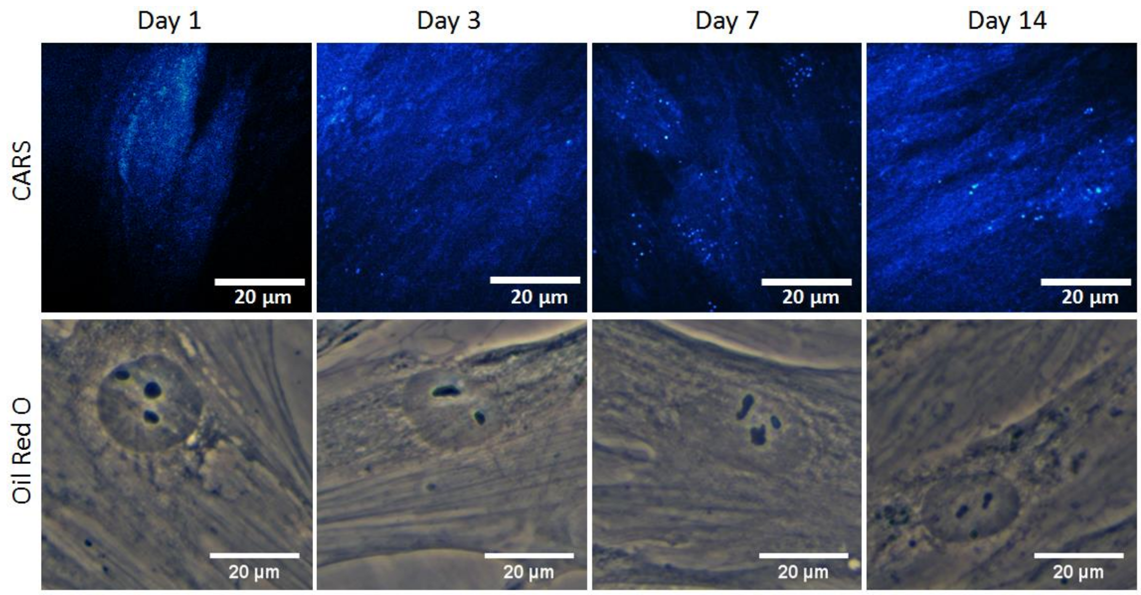

Figure S1 Comparison of label-free CARS imaging and Oil Red O staining in skeletal stem cells (SSCs) cultured in basal media for 1, 3, 7 and 14 days. Scale bars are 20 µm.

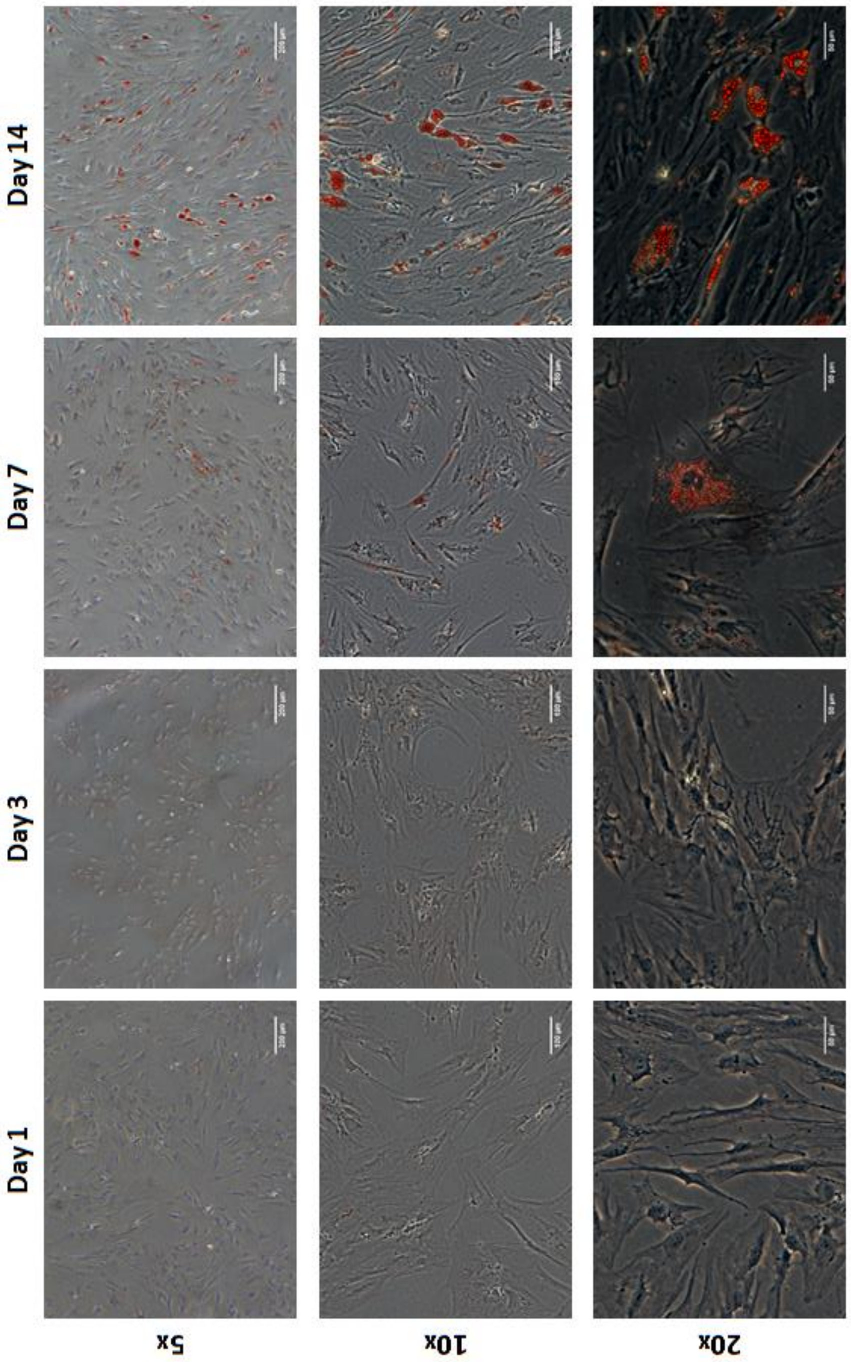

Figure S2.1 Oil Red O staining images with different objectives (5x, 10x and 20x) of SSCs cultured in adipogenic media for 1, 3, 7 and 14 days (Donor 1).

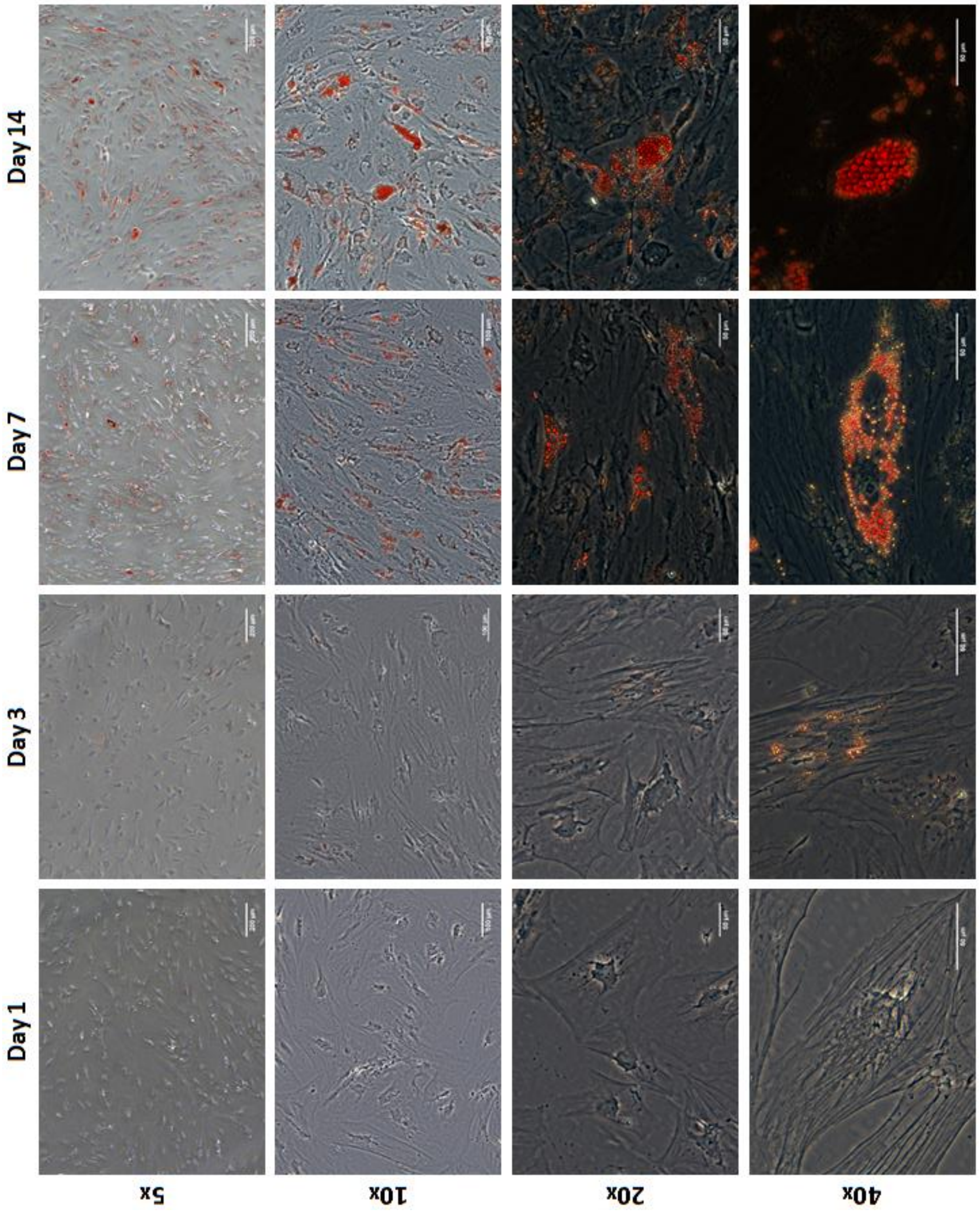

Figure S2.2 Oil Red O staining images with different objectives (5x, 10x, 20x and 40x) of SSCs cultured in adipogenic media for 1, 3, 7 and 14 days (Donor 2).

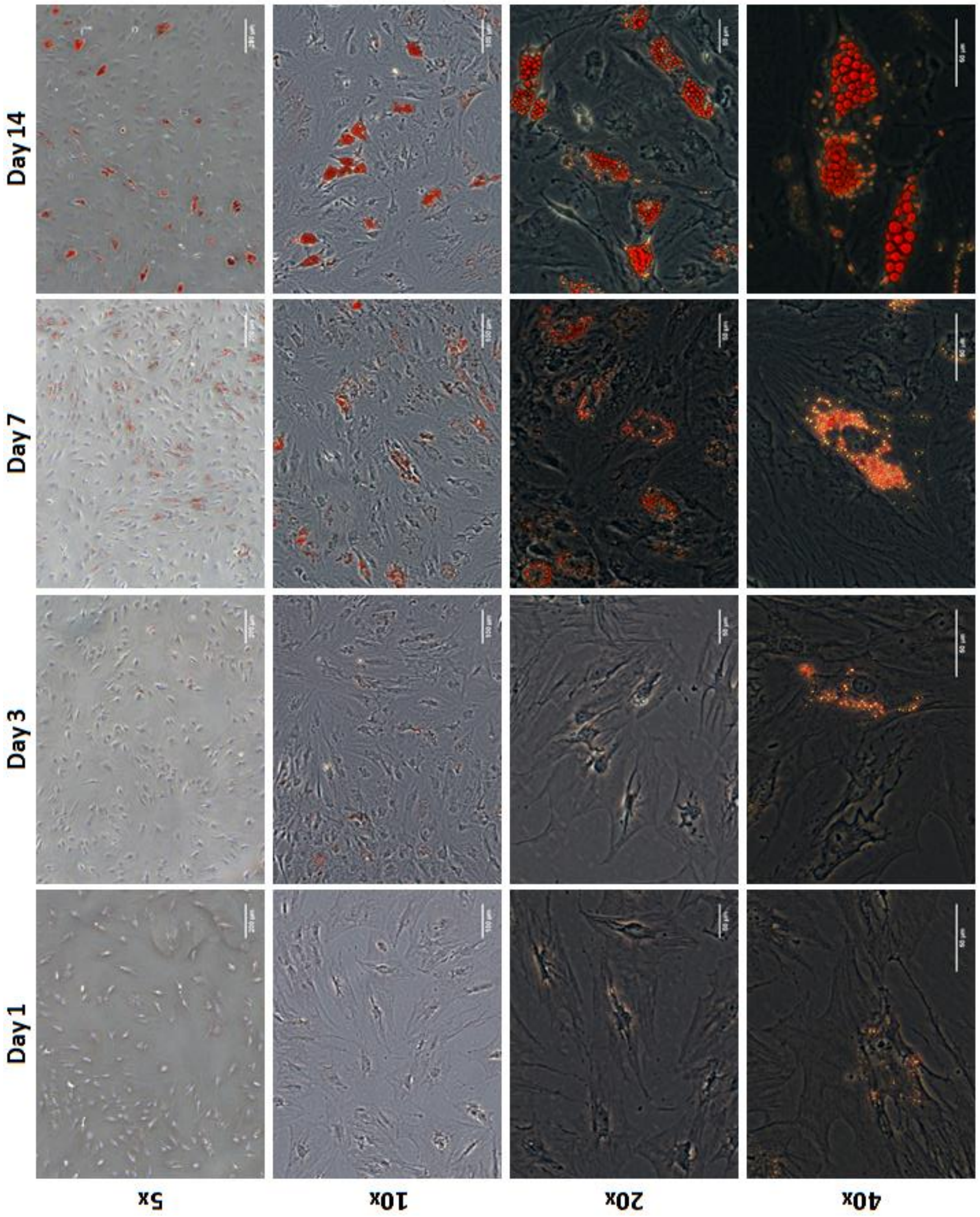

Figure S2.3 Oil Red O staining images with different objectives (5x, 10x, 20x and 40x) of SSCs cultured in adipogenic media for 1, 3, 7 and 14 days (Donor 3).
